# Immune-related gene expression in the kidneys and spleens of goslings infected with goose nephritic astrovirus

**DOI:** 10.1016/j.psj.2021.01.013

**Published:** 2021-01-19

**Authors:** Wankun Wu, Siyu Qiu, Han Huang, Rong Xu, Endong Bao, Yingjun Lv

**Affiliations:** MOE Joint International Research Laboratory of Animal Health and Food Safety, College of Veterinary Medicine, Nanjing Agricultural University, Nanjing 210095, China

**Keywords:** goose nephritic astrovirus, immune response, spleen, kidney, gosling

## Abstract

Goose nephritic astrovirus (**GNAstV**) was first isolated in 2018, causing great economic losses to the goose industry. However, little is known about host immune response to GNAstV infection. In this study, forty 2-day-old goslings were randomly divided into 2 groups: infection and negative control groups. Each gosling in the infection group was challenged with 0.5 mL GNAstV-JSHA intramuscularly, whereas the gosling in the negative control group was inoculated with the same amount of PBS. Histopathological changes and virus location in the spleen and kidney were examined, and the expression of immune-related genes was determined by qPCR at 7 and 14 d after infection. Our results showed that GNAstV infection induced degeneration and necrosis of splenic lymphocytes and renal epithelial cells, and these cells were positive for the virus. In addition, GNAstV infection induced the activation of pattern recognition receptors (RIG-I, MDA-5, and TLR3) and key adaptor molecules (MyD88, MAVS, and IRF7) in the spleen and kidney, and upregulated the gene expression of interferon-α in the spleen and antiviral proteins (MX1, OASL, and IFITM3) in the spleen and kidney. Moreover, high expression levels of interleukin **(IL)**-1β and IL-8 in the spleen and iNOS in the spleen and kidney were found. These results indicated that GNAstV infection activated host innate immune response. Furthermore, GNAstV infection increased the expression levels of CD8^+^, MHCI, and MHCII, indicating that adaptive immune response was activated. Besides, TGF-β was highly expressed in the spleen and kidney, which may be an immune evasion strategy of GNAstV to cause infection. Interestingly, both IL-1β and IL-6 mRNA levels were decreased in the kidney, which may help reduce kidney lesions. This is the first study to report changes in immune-related gene expression in response to GNAstV infection, and our results provide insights into viral pathogenesis.

## Introduction

Since 2017, several goose flocks have experienced a severe outbreak of gout in 4- to 16-day-old goslings in Eastern China, leading to major economic losses in the goose industry. The infected goslings exhibited reduced body weight, and swollen and pale kidneys, with white urates in the ureters and urate disposition on the surface of the visceral organs and articular cavity. In 2018, a new goose astrovirus was isolated from the infected goslings, and animal reproduction experiments proved that it was the main causative agent of gosling gout disease (Zhang et al., 2018a; Jin et al., 2018; Yang et al., 2018). This virus was genetically distinct from the known mamastrovirus and avastrovirus. It formed a distinct clade in accordance with the amino acid sequence of the full-length ORF2 protein (capsid protein), which is the most variable region of the genome (Zhang et al., 2018b; Yuan et al., 2019). Hence, more attention should be paid to the damage caused by the virus to goslings. In the present study, the new goose astrovirus is referred to as goose nephritic astrovirus (**GNAstV**).

Because GNAstV is a newly emerging virus, most studies have focused on its isolation, genetic analysis, and establishment of diagnostic methods (Yuan et al., 2018; Wan et al., 2019; Yin et al., 2020). However, little is known about the host immune response to GNAstV infection. Goose nephritic astrovirus mainly causes damage to the kidney, liver, and spleen in goslings, and a higher viral load has been detected in the kidney and spleen compared with that in other organs (Jin et al., 2018; Xu et al., 2019). Splenic lesions and high viral load indicate that the virus may cause damage to the immune response. The innate immune system plays an important role during virus infection by serving as the first line of defense against viral invasion (Chow J et al., 2015). Pattern recognition receptors **(PRR)** recognize viral nucleic acids and elicit an innate response, including production of cytokines, such as IL-1β, TNF-α, and IFN, and antiviral proteins such as OASL, IFITM3, and MX1. The membrane-bound Toll-like receptor 3 **(TLR3)** and the cytosolic RIG-I-like receptors which mainly contain RIG-1 and MDA-5 are the most important PRR that recognize viral RNA. The innate immune response induces signaling to elicit the adaptive immune response, which controls viral infection in subsequent phase of an infection. The adaptive immune response includes the antibody-mediated/B-cell response and the cell-mediated response, which require the involvement of CD3^+^, CD4^+^, CD8^+^, and MHC class I and II molecules. Currently, the mechanisms by which GNAstV affects host innate and adaptive immune responses are unclear. China is home to the highest goose population worldwide; therefore, investigating the goose immune response to GNAstV infection is important for elucidating the mechanism of pathogenicity and immunity of GNAstV and controlling its spread. In this study, 2-day-old goslings were infected with GNAstV. The histopathological changes and virus location, as well as immune-related gene expression in the spleen and kidney were then examined.

## Materials and methods

### Ethics Statement

All animal experiments were conducted in accordance with the Guidelines for Experimental Animals of the Ministry of Science and Technology (Beijing, China) and were approved by the Institutional Animal Care and Use Committee of Nanjing Agricultural University.

### Virus

The GNAstV-JSHA isolate (GenBank accession no. MK125058) was isolated from diseased goslings with gout (Jiangsu province, China) and kept in our laboratory. The titer of the GAstV-JSHA was 1 × 10^4.25^ 50% tissue culture infective dose (TCID50)/mL as determined by titration on goose kidney epithelial cells in accordance with the method of Reed & Muench.

### Animal Experiment

The spleen and kidney samples were collected from our previous animal experiment that was described in Xu's study (Xu et al., 2019). Briefly, 2-day-old forty goslings without GNAstV infection were randomly separated into 2 groups: infection and negative control groups. Each gosling in the infection group was challenged with 0.5 mL GNAstV-JSHA by intramuscular inoculation, whereas the gosling in negative control group was inoculated with the same amount of PBS. All goslings were monitored daily for the clinical signs. At 7 d post infection **(dpi)**, 10 goslings from each group were euthanized and the gross changes were examined, and then the kidney and spleen were collected. A portion of these tissues were fixed in 10% formaldehyde for histopathological examination, the rest were stored at −80°C for further experiments. At 14 dpi, the surviving goslings were euthanized and tissues were collected and stored as mentioned above.

### Histopathological Examination

The fixed samples were dehydrated by a series of alcohols, clarified in xylene, and embedded in paraffin. Then samples were sliced serially into 4 μm sections and stained with hematoxylin and eosin by routine methods. Stained sections were examined with a light microscope.

### Virus Location Detection by In Situ Hybridization

The in situ hybridization (**ISH**) method was performed as per the instructions of the commercial ISH kit (Boster, China), with some modifications. Before ISH was performed, the sensitivity and specificity of the sample were tested and optimized via a series of pretreatments. Briefly, deparaffinized tissue sections were pretreated with HCl (0.2 M,15 min) and proteinase K (40 μg/mL, 20 min, 37°C). Protease K was inhibited by fixation in 4% paraformaldehyde for 5 min. Then, the hybridization and posthybridization steps were performed. Probes were added to the hybridization buffer at a concentration of 0.5-2 μg/mL; the buffer containing the probes was denatured at 98°C for 10 min and immediately cooled on ice. Approximately 20 μL of the hybridization mix was pipetted onto tissue sections, covered with diethylpyrocarbonate-treated coverslips, and incubated for 16 to 20 h at 42°C. Probes that had hybridized with viral RNA were detected by using an anti-DIG antibody conjugated to 3,3-N-diaminobenzidine tertrahydrochloride. The slides were counterstained with methyl green and sealed with neutral gum. To improve sensitivity, proteinase K was used to enhance tissue permeability to facilitate the entry of probes into tissue.

### Immune Genes Analysis by Quantitative Real-Time PCR

Total RNA was extracted using Trizol (Invitrogen, CA) and reverse transcription of the RNA was conducted using the HiScript Q RT SuperMix kit (Vazyme, China). A thermocycler (AB7300; Life Technologies) was used for quantitative PCR. The Primer 5.0 software was used for the designation of the immune-related genes, as shown in Table 1. Ribonucleic acid expression was normalized by quantification of GAPDH as a housekeeping gene. Relative transcript levels were analyzed by using the 2^−ΔΔCT^ method.

**Table 1:** Primers used in the study for real-time PCR.

Primers	Accession number	Nucleotide sequence (5′-3′)	Host	Length of products(bp)
TLR3-F	MK703958.1	CAGCAAATTTAGGATGGCAAC	Anas	189
TLR3-R		ACAGATTTCCAATTGCACGTA		
MyD88-F	XM_013182382.1	CGTCTTTGATCGGGATGTCT	Anser	116
MyD88-R		CGCTTTCCAGGTAATCGTCT		
RIG-I-F	HQ829831.1	AGCACCTGACAGCCAAAT	Anser	140
RIG-I-R		AGTGCGAGTCTGTGGGTT		
MDA-5-F	JX976550.1	TGCTGTAGTGGAGGATTTG	Anser	168
MDA-5-R		CTGCTCTGTCCCAGGTTT		
MAVS-F	XM_013182243.1	GCCACATCCTGAGGAACAT	Anser	104
MAVS-R		GGTATGAAGTTCGTCCCTGTC		
IRF7-F	MG707077.1	CACCCGCCTGAAGAAGT	Anas	197
IRF7-R		GCCCGAAGCAGAGGAA		
IL-1β-F	JF505290.1	TCCGCCAGCCGCAAAGTG	Anser	136
IL-1β-R		CGCTCATCACGCAGGACA		
IL-6-F	JQ728554.1	AGATGGTGATAAATCCTGATG	Anas	150
IL-6-R		CGGTTTTCTCCATAAATGAAGT		
IL-8-F	AB213393.1	ATGAACGGCAAACTTGGGGCT	Anser	278
IL-8-R		GCCAGAATTGCCTTTACGATCA		
IL-10-F	XM_013189578.1	GGCTGCCTCCACTTGTCT	Anser	233
IL-10-R		TGGTGCTCGCTGTTCTTG		
TGF-β-F	XM_013174005.1	TCTCGGAGCAGCGGATAG	Anser	204
TGF-β-R		AGCACGGGCAATGTAAGC		
iNOS-F	U34045.1	GAACAGCCAGCTCATCCGATA	Gallus	103
iNOS-R		CCCAAGCTCAATGCACAACTT		
IFN-α-F	KF731869.1	CAGCACCACATCCACCAC	Anser	98
IFN-α-R		TACTTGTTGATGCCGAGGT		
OASL-F	KU569292.1	CAGCGTGTGGTGGTTCTC	Anser	141
OASL-R		AACCAGACGATGACATACAC		
MX1-F	KU247604.1	TTCACAGCAATGGAAAGGGA	Anser	183
MX1-R		ATTAGTGTCGGGTCTGGGA		
IFITM-3-F	KX594327.1	CCACCTGGCTTGGTCGCT	Anser	116
IFITM-3-R		CGCTGTAGTCGCCGAGGA		
MHCla-F	AY387650.1	GAGCAAGCAGGGGAAGGA	Anser	100
MHCla-R		CCGTTAGACACTGGGGTT		
MHClla-F	XM_013202352.1	CGGCCAGTTCATGTTCGAT	Anser	105
MHClla-R		AAGCTGGCAAACTTCGAGA		
CD4-F	XM_013199547.1	TTTCAACGCCACAGCAGA	Anser	127
CD4-R		GTGCCTCAACTGGATTTT		
CD8-F	KY034450.1	AGAGACGAGCAAGGAGAA	Anser	97
CD8-R		GACCAGGGCAATGAGAAG		

### Statistic Analysis

The differences between the control group and experimental group were analyzed by Student t-test. The results are expressed as the mean ± standard deviation. *P* < 0.05 was considered to indicate a statistical significance compared with the control group, and *P* < 0.01 was considered to indicate a high degree of significance compared with the control group.

## Results

### Clinical Changes

No death or obvious clinical signs were observed in the noninoculated control. However, 5 of the 20 infected goslings died during the experiment, and a reduction in body weight was observed in the GNAstV-infected goslings. At autopsy, the organs in the negative control group appeared normal, but urate disposition on the surface of the organs (liver, heart, and kidney), bile sacs, and articular cavity was detected in dead goslings. Moreover, swollen and pale kidneys with white urate in the ureters were observed in all infected goslings. The pathological changes were shown in our previous study (Xu et al., 2019). These results indicated that we successfully established an animal model of GNAstV infection in goslings.

### Histopathological Changes in the Kidney and Spleen

**Figure 1:**
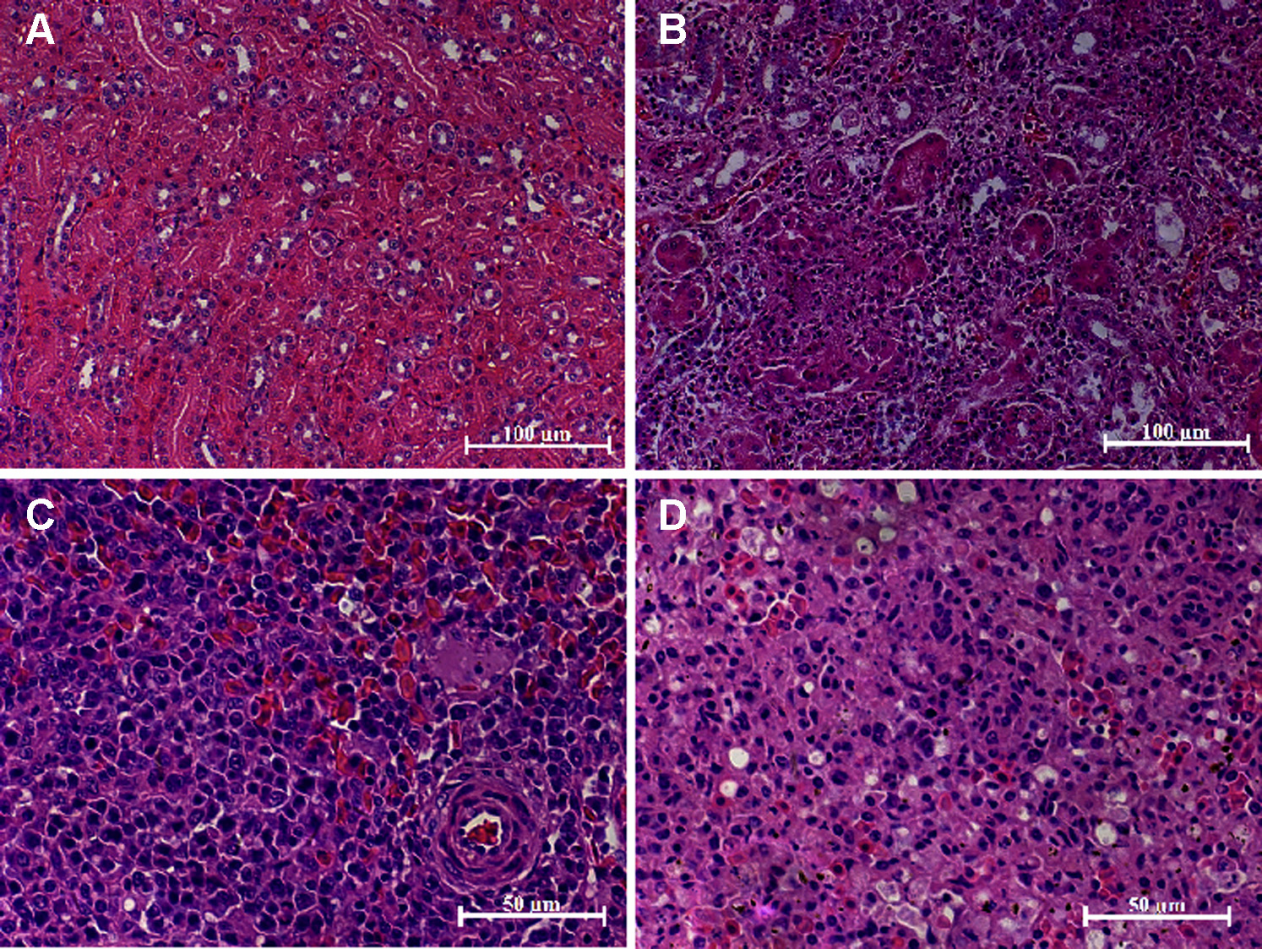
Histopathological changes in the kidney and spleen at 7 dpi after goslings experimentally infected with GNAstV. (A) The kidney of negative control goslings, (B) The kidney of infected goslings, (C) the spleen of negative control goslings, and (D) the spleen of infected goslings. Abbreviation: GNAstV, goose nephritic astrovirus.

On histopathological examination, the kidneys and spleens from the negative control goslings appeared histologically normal (Figures 1A and 1B). However, degeneration and necrosis of renal epithelial cells and inflammatory cell infiltration were observed in the kidneys of infected goslings (Figure 1C). Moreover, necrosis of splenic lymphocytes and inflammatory cell infiltration were also found in the spleens (Figure 1D).

### Virus Location in the Kidney and Spleen

**Figure 2:**
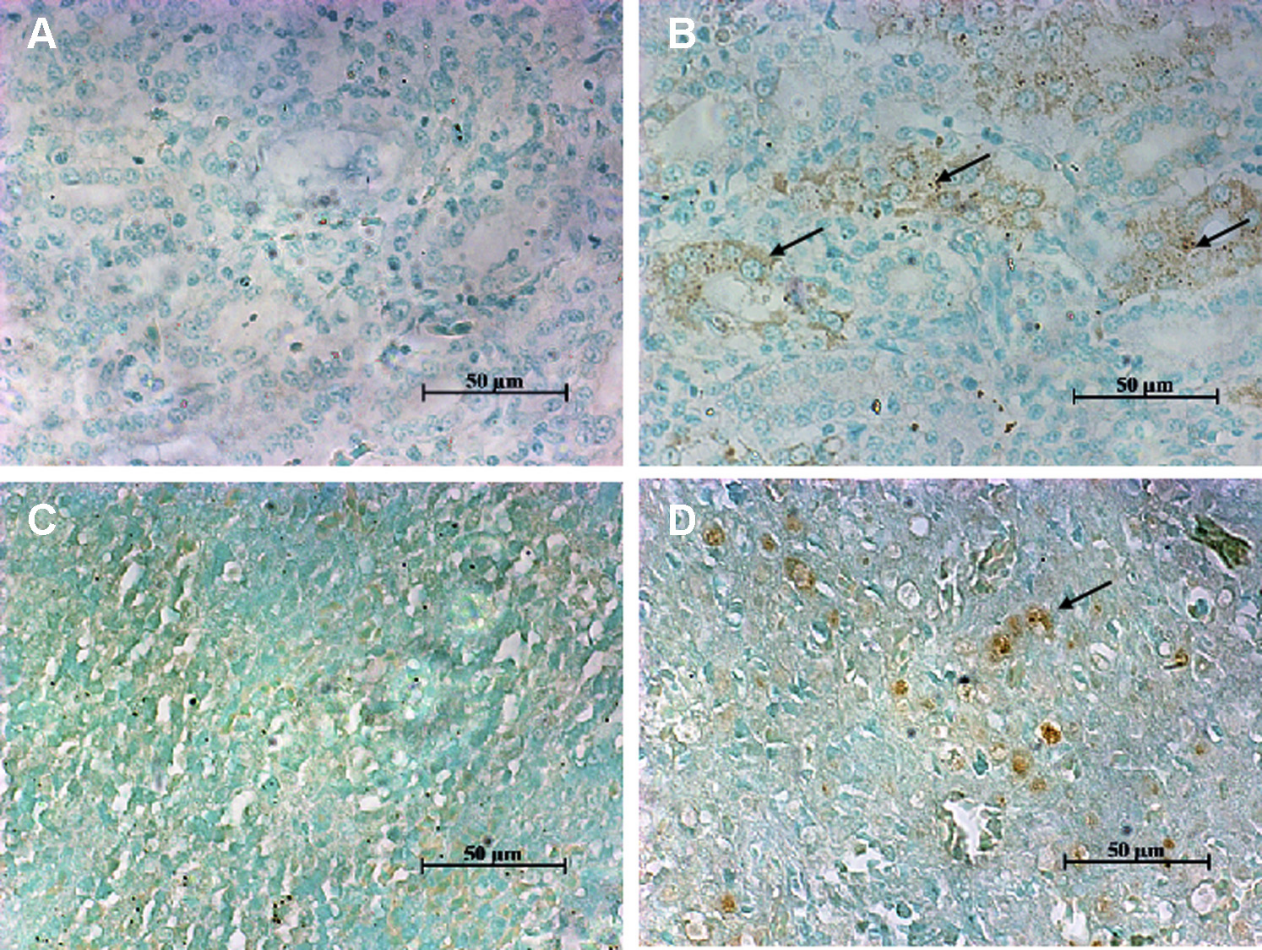
Virus location in the kidney and spleen at 7 dpi after goslings experimentally infected with GNAstV. Abbreviation: GNAstV, goose nephritic astrovirus.

Goose nephritic astrovirus location in the kidneys and spleens was examined using ISH. As shown in Figure 2, several brown particles were detected in the cytoplasm of renal tubular epithelial cells, but no brown particles were observed in the glomeruli of the kidney. Few brown particles were detected in the splenic lymphocytes and macrophage cells of the spleen. These results indicate that GNAstV can infect renal tubular epithelial cells and splenic lymphocytes.

### Expression of PRR and Adapter Molecules in the Spleen and Kidney of GNAstV-Infected Goslings

**Figure 3:**
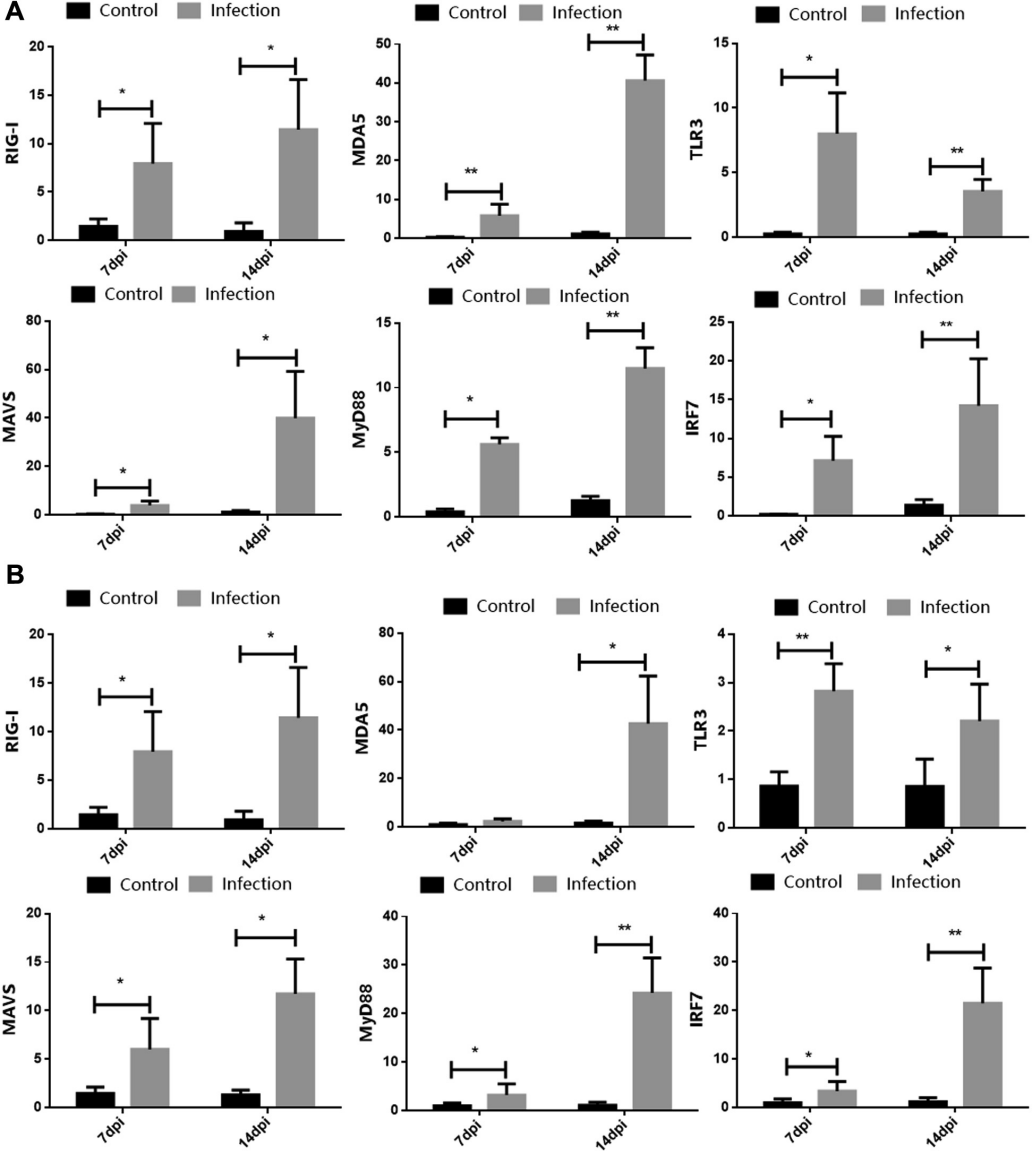
Changes in the mRNA levels of RIG-I, MDA-5, TLR3, MAVS, MyD88, and IRF7 in the spleen (A) and kidney (B) at 7 and 14 dpi after inoculation with GNAstV. Values are expressed as mean ± SD, n = 10. ∗*P* < 0.05; ∗∗*P* < 0.01. Abbreviation: GNAstV, goose nephritic astrovirus.

The expression of PRR (RIG-1, MDA-5, and TLR3) and key adapter molecules (MAVS, MyD88, and IRF7) in the spleens and kidneys was determined by qPCR as shown in Figure 3. The mRNA level of the 3 PRR in the spleens was significantly increased in the infected group at 7 and 14 dpi compared with the negative control group (*P* < 0.05). The RIG-1 and TLR3 mRNA levels in the kidneys were also increased in the infected group at 7 and 14 dpi compared with that in the negative control group (*P* < 0.01). The MDA-5 mRNA level was higher in the infected group at 14 dpi, but no difference in MDA-5 mRNA expression was observed between the infected and negative control groups at 7 dpi (*P* > 0.05). Accordingly, the expression of the adapter molecules in the infected group at 7 and 14 dpi was significantly higher than that in the control group (*P* < 0.05). These results indicate that GNAstV infection activates host innate immune response.

### Cytokine Expression in the Spleen and Kidney of GNAstV-Infected Goslings

**Figure 4:**
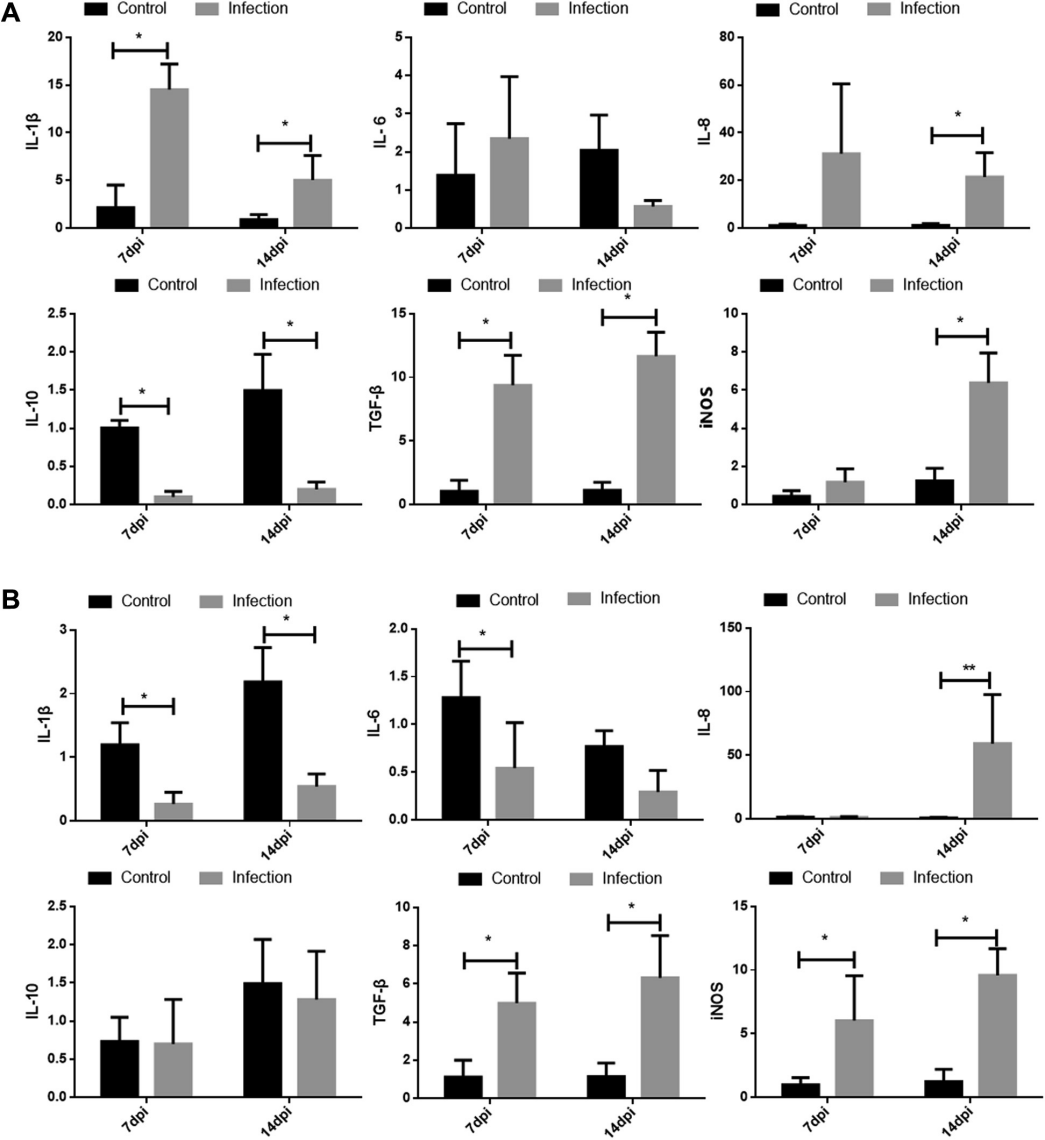
Changes in the mRNA levels of IL-1β, IL-6, IL-8, IL-10, TGF-β, and iNOS in the spleen (A) and kidney (B) at 7 and 14 dpi after inoculation with GNAstV. Values are expressed as mean ± SD, n = 10. ∗*P* < 0.05; ∗∗*P* < 0.01. Abbreviation: GNAstV, goose nephritic astrovirus.

The expressions of the cytokine, IL-1β, IL-6, IL-8, IL-10, TGF-β, and iNOS were determined by qPCR as shown in Figure 4. In the spleen, the mRNA levels of IL-1β and TGF-β in the infection group at 7 and 14 dpi were obviously higher than those in the negative control group (*P* < 0.01). Furthermore, the IL-8 and iNOS mRNA levels in the infection group at 14 dpi were higher (*P* < 0.05), but no differences in the levels were observed between the infection and negative control groups at 7 dpi (*P* > 0.05). Moreover, no difference in IL-6 mRNA expression at 7 and 14 dpi was observed between the infection and control groups (*P* > 0.05). In the kidneys, the mRNA levels of IL-1β at 7 and 14 dpi and IL-6 at 7 dpi were lower in the infection group than those in the negative control group (*P* < 0.05), whereas the TGF-β and iNOS mRNA levels at 7 and 14 dpi and the IL-8 mRNA level at 14 dpi were higher in the infection group than those in the negative control group (*P* < 0.05). No difference in IL-10 expression was detected between the infection and control groups (*P* > 0.05).

### Expression of Antiviral Proteins in the Spleen and Kidney of GNAstV-Infected Goslings

**Figure 5:**
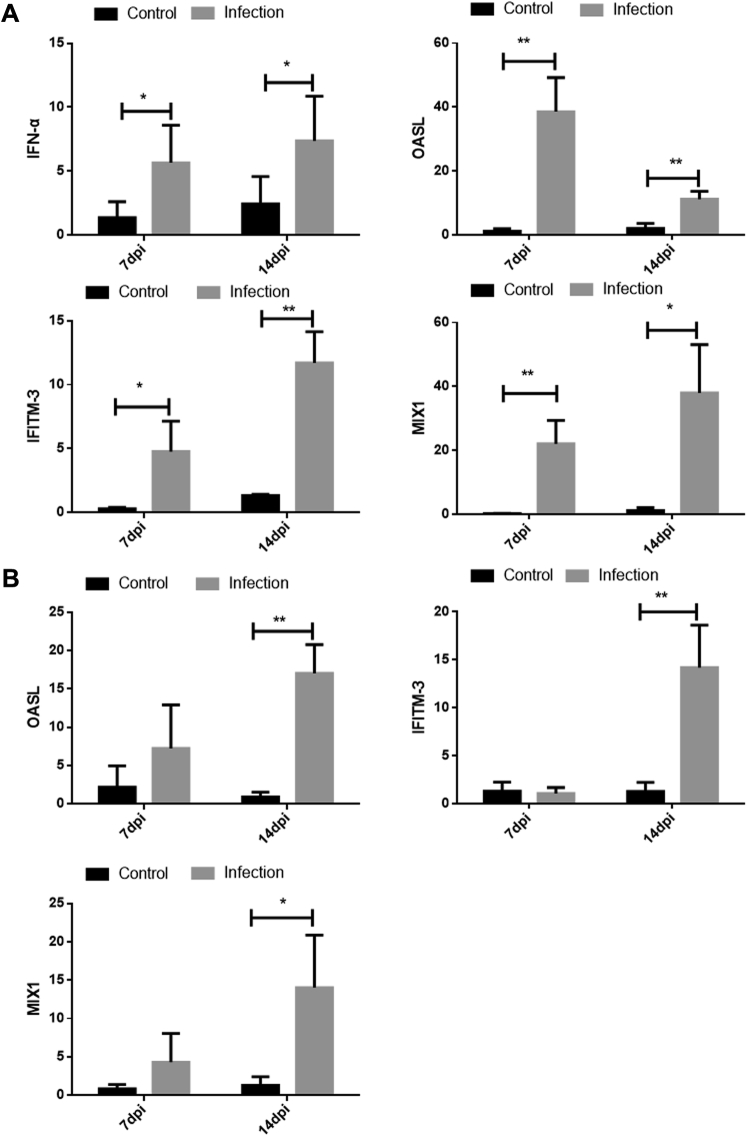
Changes in the mRNA level of IFN-α in spleen and the mRNA levels of OASL, MX1 and IFITM3 in the spleen (A) and kidney (B) at 7 and 14 dpi after inoculation with GNAstV. Values are expressed as mean ± SD, n = 10. ∗*P* < 0.05; ∗∗*P* < 0.01. Abbreviation: GNAstV, goose nephritic astrovirus.

The mRNA level of IFN-α in the spleen and the expression of antiviral proteins (OASL, IFITM3, and MX1) in the kidney and spleen were determined as shown in Figure 5. In the spleen, the mRNA levels of IFN-α, OASL, IFITM3, and MX1 at 7 and 14 dpi were significantly higher in the infected group than those in the negative control group (*P* < 0.05). In the kidney, the mRNA levels of OASL, IFITM3, and MX1 at 14 dpi were obviously higher in the infected group than in the negative control group (*P* < 0.05); however, no significant differences in levels were observed between the infection and negative control group at 7 dpi (*P* > 0.05).

### Expression of CD4^+^, CD8^+^, and MHC Class I and II Molecules in the Spleen of GNAstV-Infected Goslings

**Figure 6:**
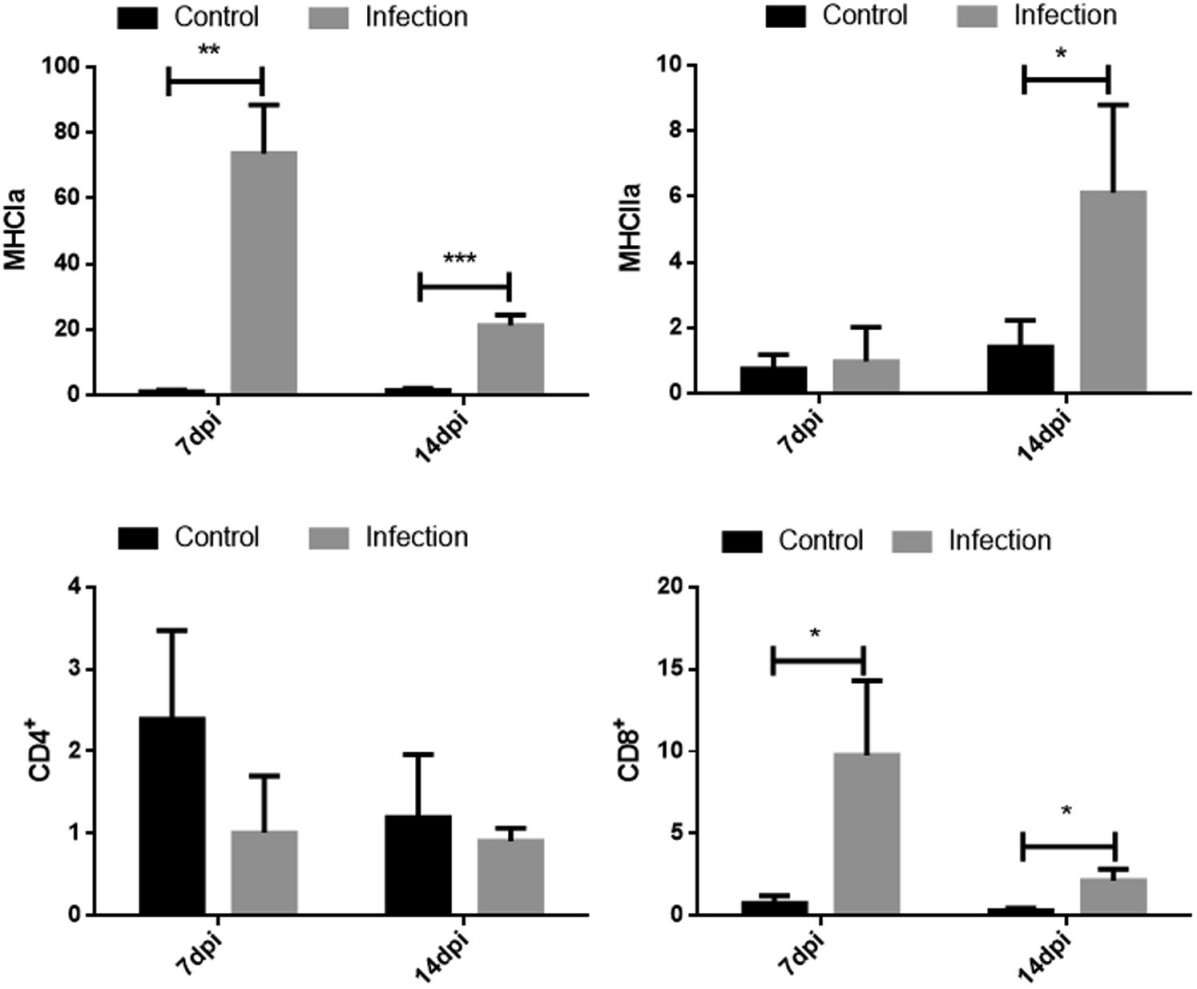
Changes in the mRNA levels of CD4^+^, CD8^+^, MHC class I and II molecules in the spleen at 7 and 14 dpi after inoculation with GNAstV. Values are expressed as mean ± SD, n = 10. ∗*P* < 0.05; ∗∗*P* < 0.01; ∗∗∗*P* < 0.001. Abbreviation: GNAstV, goose nephritic astrovirus.

The expression of genes associated with antigen presentation (CD4^+^, CD8^+^, MHC class I and II molecules) in the spleens was determined by qPCR as shown in Figure 6. The expression levels of MHC I at 7 and 14 dpi were significantly higher in the infected group than those in the negative control group (*P* < 0.05). The expression levels of MHC II at 14 dpi were obviously higher in the infected group than those in the negative control group (*P* < 0.05); however, no difference in levels was observed between the 2 groups at 7 dpi (*P* > 0.05). The CD8^+^ mRNA levels at 7 and 14 dpi were significantly higher in the infected group than those in the negative control group (*P* < 0.05), but no changes in CD4^+^ mRNA levels at 7 and 14 dpi were found between the 2 groups (*P* > 0.05).

## Discussion

Goose nephritic astrovirus is a newly isolated virus that can cause visceral gout and death of goslings, leading to great damage to goose flocks. To date, there are no vaccines and therapeutic agents for its treatment. Hence, it is necessary to investigate host immune response to the virus to understand its pathogenesis and develop vaccines and therapies. In this study, we successfully established an animal model in goslings by intramuscular inoculation with GNAstV-JSHA. The location of the virus in splenic lymphocytes and macrophage and necrosis of splenic lymphocytes indicate that GNAstV can infect the spleen and induce damage to the host immune system. However, until now, there have been no reports on the immunological response of goslings infected with GNAstV. In addition, a high viral titer was observed in the renal epithelial cells, and this may contribute to kidney damage and uric acid excretion disorder.

The innate immune system is the first line of defense against an invading pathogen. In our study, GNAstV infection caused increase of TLR3, RIG-1, and MDA-5 mRNA expression in the spleen and kidney, indicating that the innate immune system was activated which may contribute to inhibition of viral invasion. The upregulation of key adaptor molecules (MyD88 and IRF7) further confirmed these results. IFN are produced after the activation of PRR, which play a critical role in viral suppression via induction of several IFN-stimulated genes whose products have direct antiviral activity. In our study, the expression of IFN-α in the spleen and the expression of antiviral proteins (OASL, MX1, and IFITM3) in the spleen and kidney were increased after GNAstrV infection. Increased production of antiviral proteins may be useful for the inhibition of viral invasion. In accordance with this, our results showed that TLR3, RIG-1, MDA-5, and IRF7 were activated during GNAstV infection. A previous study also demonstrated that the type I IFN system limits human astrovirus replication in vitro and in vivo and provides protection against astrovirus-induced barrier permeability (Marvin et al., 2015). However, the finding for TLR3 and OASL were different from hose for the other PRR and antiviral proteins in the spleen, as the expression of both TLR3 and OASL decreased at 14 dpi compared with 7 dpi. This might be related to negative feedback regulation or reduction of virus load. Accordingly, it was reported that virus shedding at 15 dpi was lower than that at 7 dpi (An et al., 2020).

Cytokines production is a host immune response to viral infection, and plays an important role in immune regulation. IL-1β is a proinflammatory cytokine, which affects the differentiation of T cells and enables the transmigration of immunocompetent cells to infection sites. The IL-1β mRNA level was significantly increased in the spleen after GNAstV infection, which may initially inhibit GNAstV infection, but excess production of IL-1β may cause splenic lesions. The decline of IL-1β at 14 dpi may be a result of body regulatory mechanisms against excess proinflammatory cytokine production. Interestingly, the mRNA level of IL-1β in the kidney was decreased in the infected group. A lower level of this proinflammatory cytokine may help alleviate kidney damage. Previous studies reported that human astrovirus and turkey astrovirus type 2 **(TAStV-2)** infections were not associated with inflammation and caused few lesions in the intestinal villi (Koci et al., 2003; Sebire et al., 2004), suggesting that this may be a characteristic of astrovirus infection in epithelial cells. IL-6 is also an inflammatory cytokine that induces antiviral effects via an efficient immune response, but is also involved in processes that lead to vascular damage, inflammation of the vascular wall, and thrombosis. In our study, no significant change in IL-6 was found in the spleen after GNAstV infection; this might be related to large differences between individual goslings. However, IL-6 expression was decreased in the kidney, similar to the observation for IL-1β; this may also help alleviate kidney damage. IL-8 is a mediator responsible for the recruitment of neutrophils that participate in the local inflammatory infiltrate. In this study, IL-8 was upregulated in the spleen and kidney, and it may have thus contributed to the inflammation. IL-10 is an anti-inflammatory cytokine that can inhibit proinflammatory cytokine production. In this study, GNAstV caused an obvious decrease in IL-10 levels in the spleen; this might be an indication of severe inflammation in the spleen and one of the causes of spleen damage. Infection with other viruses infection, such as Japanese encephalitis virus and Zika virus, has also been shown to result in a decrease in IL-10 expression (Swarup et al., 2007; Abreu 2019), but the exactly mechanism underlying GNAstrV infection needs further study. TGF-β is an immunosuppressive cytokine. Thus, the increase in TGF-β levels in the spleen and kidney indicates that GNAstV infection may induce immune suppression. Furthermore, it was reported that TAStV-2 infection increased the serum level of TGF-β in turkeys (Koci et al., 2003). Increased TGF-β production has been shown to suppress immune response and enhance viral replication (Letterio and Roberts, 1998). Hence, increased TGF-β production might contribute to GNAstV replication by inhibiting host immune response. iNOS is an important component of the innate immune response and plays a key role in controlling viral infections. In our study, the findings indicated that GNAstV infection obviously upregulated iNOS expression. High iNOS production may enable the host to resist viral infection. Our results were in accordance with those of previous studies, which showed that TAStV-2 infection induces iNOS production, which limits astrovirus replication (Qureshi et al., 2001; Koci et al., 2004; Meyerhoff et al., 2012).

The adaptive immune system plays a key role in controlling viral infections. In our study, GNAstV infection increased the mRNA level of MHC Iα, MHC Iiα, and CD8^+^, and this indicates that humoral and cellular immune responses were activated. Previously, it was reported that human astrovirus infection evokes antibody production, which can neutralize the virus (Kurtz and Lee, 1978). However, no significant changes were found in CD4^+^ mRNA expression in the study. Considering that CD4^+^ T cells are essential for B-cell maturation and antibody specificity, it is possible that GNAstV could not induce a strong humoral immune response. This may explain why no obvious increase in MHC IIα expression was observed at 7 dpi. In addition, the mRNA levels of CD8^+^ and MHC Iα at 14 dpi decreased compared with 7 dpi. This may be related to the reduction of virus load, as mentioned above. In this study, the GNAstV antibody was not detected because no commercial kit is available. Hence, the role of antibodies in GNAstV infection needs to be further investigated.

In conclusion, our data revealed the immune response patterns in the spleen and kidney of GNAstV-infected goslings. On GNAstV infection, genes related to innate and adaptive immunity were activated. However, the production of cytokines and antiviral proteins did not protect the goslings against the disease. This is the first study to report the changes in immune-related gene expressions in response to GNAstV infection, which may further help elucidate the molecular mechanisms of host GNAstV interactions.
